# An impedimetric approach for determination of ammonium using silver/poly-1-aminoanthraquinone/carbon paste electrode

**DOI:** 10.1038/s41598-024-68321-x

**Published:** 2024-08-09

**Authors:** Mahmoud Fatehy Altahan, Amr Mohamed Beltagi, Magdi Abdel-Azzem, Mona A. El-Attar

**Affiliations:** 1 Central Laboratory for Environmental Quality Monitoring, National Water Research Center, El-Qanater El-Khairia, 13621 Egypt; 2 Chemistry Department, Faculty of Science, Kafrelsheikh University, Kafrelsheikh, 33516 Egypt; 3 Chemistry Department, Faculty of Science, Menoufia University, Shibin El-Kom, 32511 Egypt; 4 High Institute of Engineering & Technology (THIET), Tanta, 31739 Egypt

**Keywords:** Impedimetric sensor, Silver-based sensor, Ammonium sensor, Poly-aminoanthraquinone based sensor, Electrochemical impedance spectroscopy, Chemistry, Analytical chemistry, Sensors

## Abstract

Here, we present the electrochemical determination of ammonium in water samples, emphasizing the importance of accurate and precise assessment of its concentration. The modified electrode used in this study was fabricated through the anodic polymerization of 1-aminoanthraquinone (1-AAQ) and deposition of silver particles into a carbon paste electrode. The fabrication process involved cyclic voltammetry in a 0.1 M HCl solution, followed by the application of a potential of 0.2 V for 75 s. The resulting Ag/poly-1-AAQ/CPE exhibited remarkable electrochemical properties, as confirmed by scanning electron spectroscopy (SEM), energy-dispersive X-ray analysis (EDX), and elemental mapping. The successful deposition of silver at percentages of 12.07% on Ag/CPE and 0.75% on Ag/poly-1-AAQ/CPE was observed. The Ag/poly-1-AAQ/CPE was employed for impedimetric determination of ammonium in a solution of 0.1 M Na_2_SO_4_. The charge transfer resistance) output from the fitting of the experimental impedimetric data of ammonium determination exhibited good linearity over a concentration range of 5 µM to 200 µM NH_4_^+^, with a detection limit of 3.3 µM NH_4_^+^. The precision of the modified electrode over ten replicate measurements were conducted at three concentration levels (a low of 5 µM NH_4_^+^, a medium of 50 µM NH_4_^+^, and a high of 200 µM NH_4_^+^). The obtained relative standard deviation (RSD) values of 18%, 12% and 7%, respectively, indicating good precision.

## Introduction

Ammonium, an onium cation containing nitrogen, plays a crucial role in the nitrogen cycle within natural water bodies. It is essential for the growth and development of aquatic ecosystems, serving as a vital nutrient source for primary production processes. However, when excessive amounts of ammonium enter aquatic environments, it can give rise to various issues including excessive phytoplankton growth, freshwater pollution, eutrophication, and the proliferation of toxin-producing algae^1^. Therefore, it is of utmost importance to accurately determine the levels of ammonium in natural water^2^. This precise assessment is vital for evaluating water quality, conducting environmental assessments, and preserving ecological health. Among the conventional techniques utilized for ammonium determination, spectrophotometric methods such as the Nessler's reagent assay and the Indophenol blue (IPB) reagent method^3^. The Nessler's reagent-based method detects ammonium through the formation of a yellow–brown complex but it can be affected by interference from calcium and magnesium ions^4^. The IPB method generates the Indophenol blue dye by reacting ammonium with hypochlorite and alkaline phenol^5^. On the other hand, fluorometric methods specifically the ortho-phthaldehyde OPA assayutilizes amino acids reactions to form a fluorescent dye^6^.

Despite the sensitivity of wet-chemical methods for ammonium determination, they suffer from reagent consumption over time, making them less suitable for long-term deployment in on-site applications and sensors. This has prompted the application of electrochemical methods, which offer advantages such as ease of operation, simplicity, and suitability for long-term deployment.

Among the electrochemical methods, potentiometric ion-selective electrodes (ISEs) provide several benefits, including easy operation, fast response time, and the potential for miniaturization, enabling in-situ analysis and a wide dynamic response range^7,8^. However, the application of ISE for ammonium determination in seawater is limited due to the presence of high chloride ion content, which poses challenges for accurate measurements^9^.

Recently, we have published reports on the determination of ammonium based on voltammetric analysis. This technique involves the complexation of ammonia with silver in Tollen's reagent, which can then be detected electrochemically^10^. Square wave voltammetry offers various advantages, such as reducing the capacitive current and enhancing the sensitivity of the electrochemical analysis approach^10^.

Electrochemical impedance spectroscopy (EIS) presents an excellent approach for the electrochemical determination of ammonium. EIS offers the ability to analyze the kinetics, electrochemical charge transfer properties, and electrical characteristics of the system under investigation^11,12^. This technique has various advantages for the development of sensors, including monitoring the changes in the sensor surface over time and providing enhanced sensitivity for monitoring analyte concentrations by considering the electrochemical properties of the electrolyte-sensor interface.

Carbon paste electrodes (CPEs) are popular due to their advantages: inertness, robustness, renewability, stable response, low resistance, versatility, and eco-friendliness. Yet, traditional CPEs have drawbacks: lower sensitivity, reproducibility, slower kinetics, limited stability, and increased over-potential for electro-catalysis^13^.

In this study, we focus on the enhancement of CPE by the addition of a conductive polymer, specifically poly-1-AAQ, due to its remarkable chelation properties and the presence of high conjugation within its three aromatic rings. Additionally, the polymer features two quinone groups and an amino group, providing abundant active sites that make it highly suitable for sensor development^14^. By depositing silver on the electrode, there is an opportunity for chelation with ammonium in Tollen's reagent, which can then be detected electrochemically. This is particularly advantageous as ammonium itself is electrochemically inactive.

The determination of ammonium on Ag/poly-1-AAQ/CPE was carried out using electrochemical impedance spectroscopy. The linear range of ammonium concentration was determined, and precision was evaluated for low, medium, and high ranges of ammonium concentration. The implementation of EIS for the determination of ammonium concentrations allows for the potential integration of impedimetric analysis into a prototype for direct quantification of ammonium and follow our progress in developments of deployable sensors for chemicals in the aquatic environment^10,14–23^.

## Materials and methods

### Materials

The chemicals 1-aminoanthraquinone (AAQ) (C_14_H_9_NO_2,_ 97%), graphite (particle size < 20 μm), silver nitrate (AgNO_3_, ACS reagent, ≥ 99.0%), and puriss. grade paraffin oil were obtained from Sigma Aldrich, U.S. Ammonium Chloride (NH_4_Cl, 99.998%, trace metals basis) and Sodium sulphate (Na_2_SO_4_, ACS reagent, ≥ 99.0%, anhydrous) were obtained from Merck, U.S.

### Instruments

The electrochemical experiments were performed using a PalmSens 4 Potentiostat/Galvanostat/Impedance Analyzer (PalmSens BV, Randhoeve 221, 3995 GA Houten, The Netherlands), which was computer controlled. The PSTrace 5 software version 5.9 (PalmSens BV) was utilized for controlling the instrument. The experiments employed a three-electrode system. To analyze the EIS (Electrochemical Impedance Spectroscopy) data and select the appropriate equivalent circuit, the Biologic EC-LAB software was employed. The data was then presented using Python software version 3.4.3. The three-electrode system consisted of a CPE holder (BASi, U.S.) as the working electrode, an Ag/AgCl reference electrode (3M KCl), and a platinum wire counter electrode. All EIS measurements were conducted in the frequency range from 10 kHz to 0.1 Hz.

The characterization of the modified electrodes was performed using SEM (JEOL, Japan) connected with an EDX instrument (JSM IT 100, Japan) for SEM imaging, EDX analysis, and elemental mapping analysis. SEM analysis was conducted at the Nano Science & Technology Institute, Kafrelsheikh University, Egypt.

### Methods

The composition for preparing Ag/poly-1-AAQ/CPE involved mixing 0.59 g of graphite powder, 0.01 g of AgNO_3_, 0.1 g of 1-aminoanthraquinone, and 0.3 g of paraffin oil to form a total 1 g of modified carbon paste. The mixing process was carried out in a mortar with a pestle for 10 min until a uniform paste was obtained. The paste was then packed into the electrode holder by pressing it at the end of the holder and further polishing on a filter paper to remove any residues. The electrode was rinsed with deionized water and prepared for the activation step.

**Figure 1:**
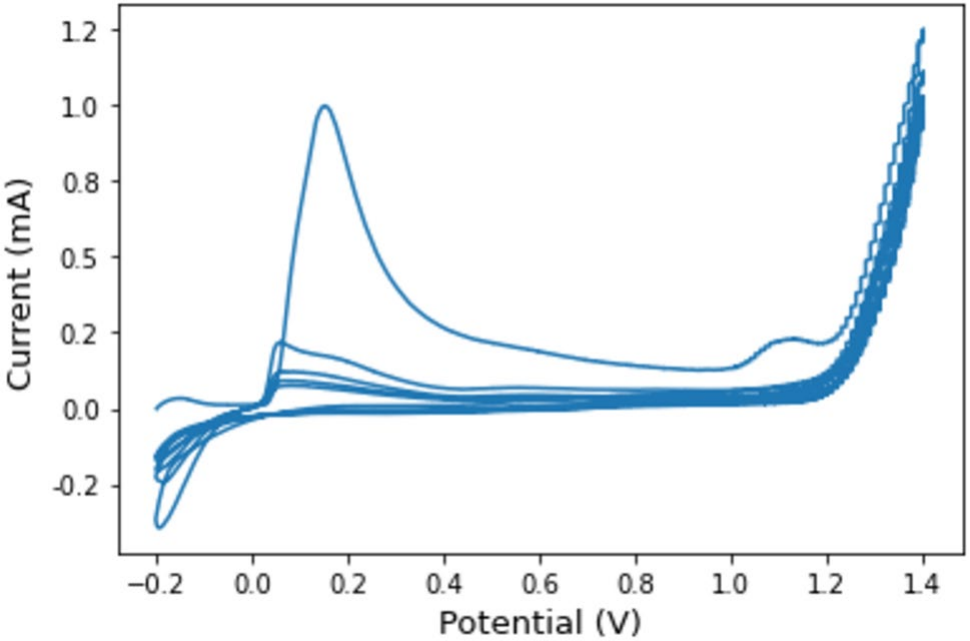
Cyclic voltammograms were recorded for the electrode modified with Ag/poly-1-AAQ/CPEin a solution of 0.1 M HCl. The measurements were performed over 5 sampling cycles at a sampling rate of were V s^−1^, within a potential range from − 0.2 V to 1.4 V, with a switching potential at − 0.2 V. Prior to the cyclic voltammetry measurements, a fixed potential of 0.75 V was applied for 2 min with stirring.

The activation process involved applying a potential of + 0.75 V for 120 s under stirring at 400 rpm. Electrochemical polymerization was then carried out via cyclic voltammetry within a potential range of − 0.2 V to 1.4 V in 0.1 M HCl for five scanning cycles as shown in Fig. 1.

For Ag/CPE, the preparation involved mixing 0.69 g of graphite powder, 0.01 g of AgNO_3_, and 0.3 g of paraffin oil to form a total 1 g of the paste. The same mixing process was followed, and the resulting uniform paste was pressed into the electrode holder. Activation was performed only through the applied potential step.

For Ag/poly-1-AAQ/CPE, 1 g of paste was formed by mixing 0.59 g of graphite powder, 0.01 g of AgNO_3_, 0.1 g of 1-AAQ, and 0.3 g of paraffin oil. The paste was pressed into the electrode holder, and activation was performed via cyclic voltammetry.

For CPE, 1 g of paste was prepared by mixing 0.7 g of graphite powder and 0.3 g of paraffin oil, following the same process for preparing and pressing the electrode.

Impedimetric analysis was conducted in a solution of 0.1 M Na_2_SO_4_. A stock solution of ammonium was prepared by dissolving ammonium chloride at a concentration of 1 mM in deionized water. Different concentrations of NH_4_^+^ were obtained by taking different aliquots and adding them to a solution of 0.1 M Na_2_SO_4_. Before the analysis, all the solutions were deoxygenated by bubbling with pure nitrogen.

## Results and discussion

### Characterization of modified electrodes

**Figure 2:**
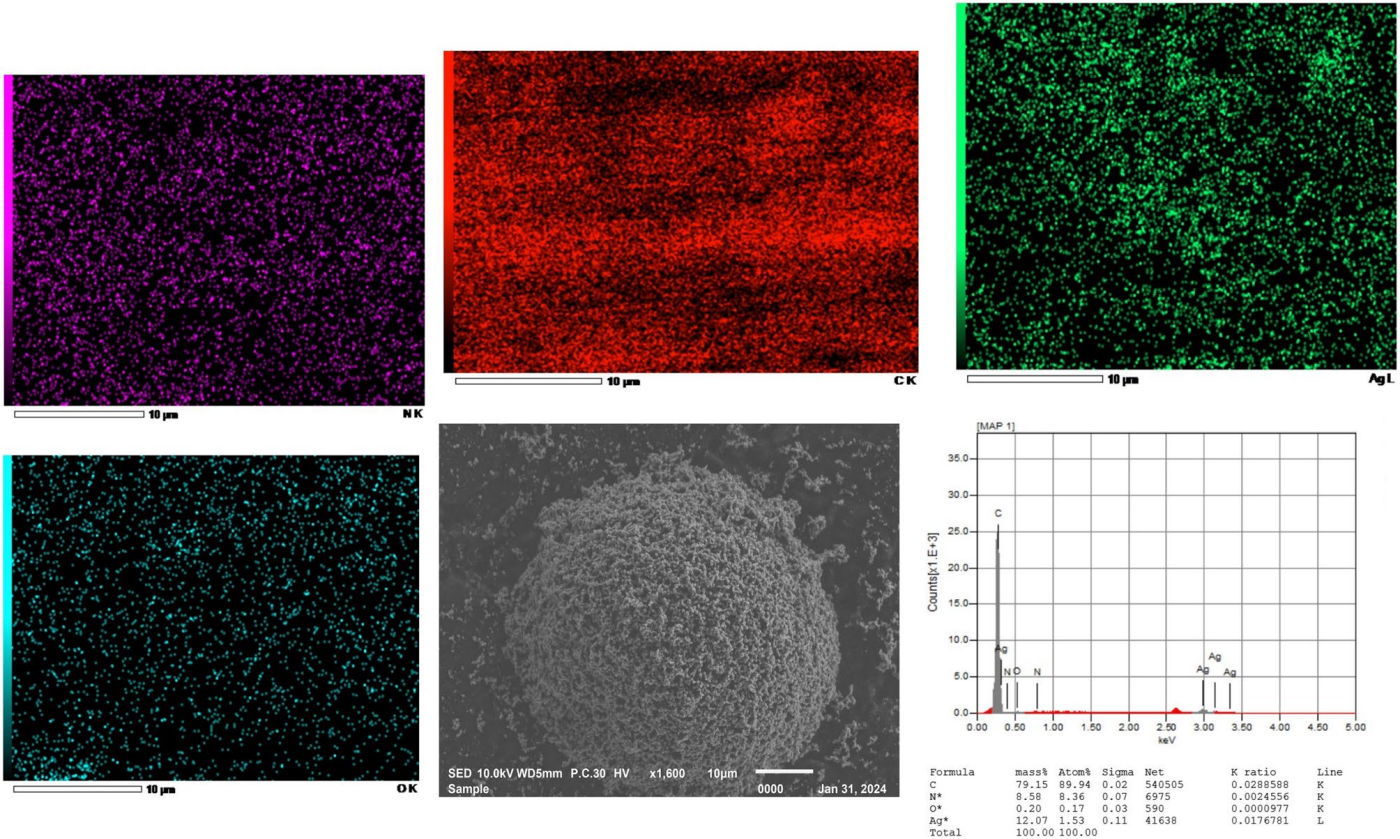
SEM image of the Ag/CPE modified electrode at a scale of 10 µm, showing the distribution of C, O, N, and Ag atoms. Elemental analysis was performed using EDX analysis, revealing a mass percentage of 79.15% for C, 8.58% for N, 0.2% for O, and 12.07% for Ag on the electrode surface.

**Figure 3:**
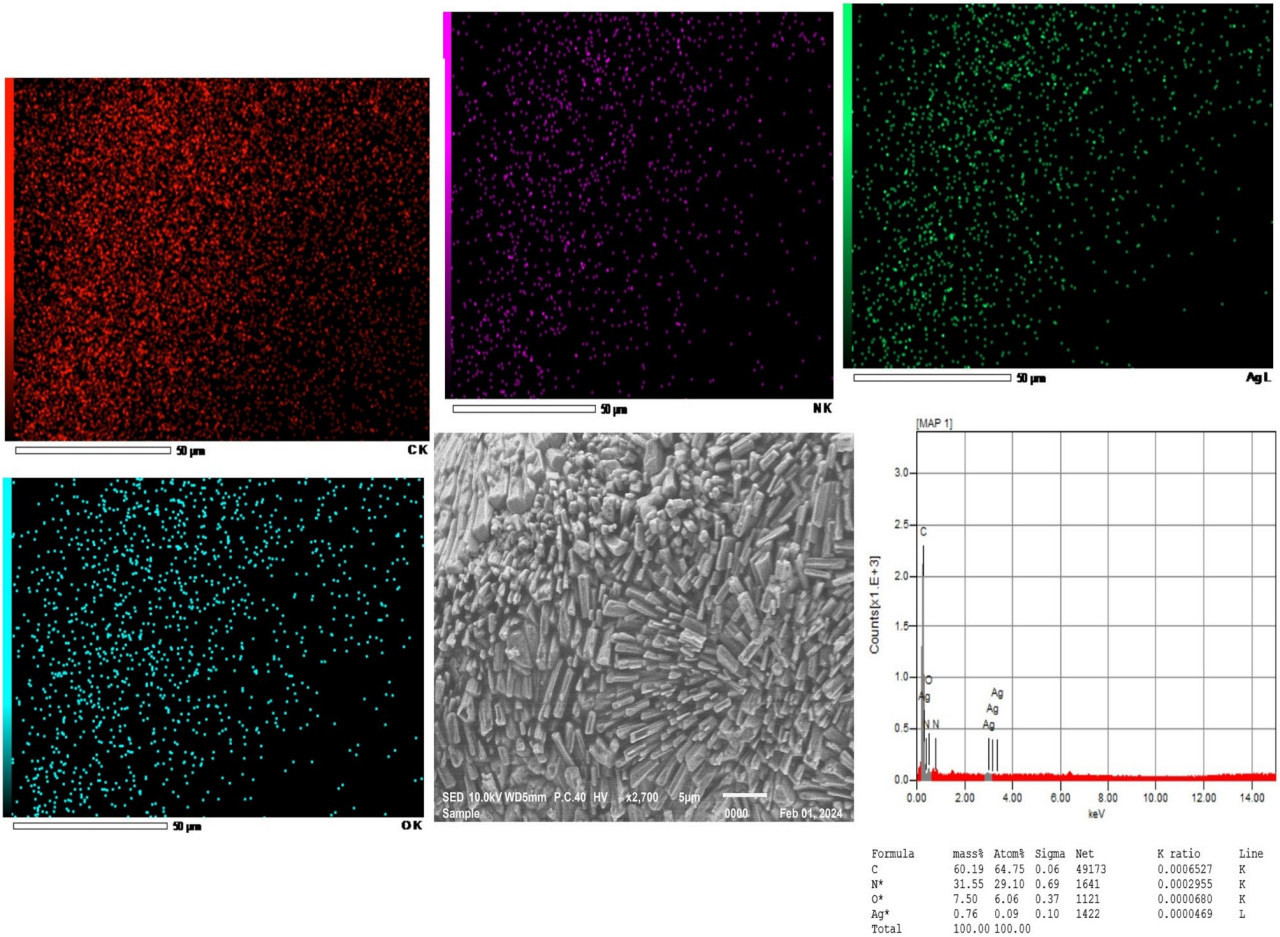
SEM image of the Ag/poly-1-AAQ/CPE modified electrode at a scale of 5 µm, illustrating the distribution of C, O, N, and Ag atoms. Elemental analysis through energy-dispersive X-ray analysis reveals a mass percentage of 60.19% for C, 31.55% for N, 7.50% for O, and 0.76% for Ag on the surface of the electrode.

The modified electrodes, Ag/CPE and Ag/poly-1-AAQ/CPE, underwent characterization using SEM to analyse their structure and morphology. Additionally, the surface structures of these electrodes were further examined using EDX and elemental mapping analysis, as represented in Figs. 2 and 3, respectively. The SEM image of Ag/CPE, as shown in Fig. 2, exhibited a cluster of silver particles that had accumulated following the application of a potential of 0.75 V during the activation step. The cluster predominantly consisted of carbon atoms, with smaller quantities of nitrogen and oxygen, as confirmed by both EDX and the elemental mapping analysis. Interestingly, the mass percentage analysis revealed that silver atoms constituted 12.07% of the accumulated cluster. These findings affirm the successful deposition of silver atoms, which are now accessible for chelation with ammonium ions in the Tollen's complex reagent, resulting in the formation of a diammonium silver complex. Furthermore, these results provide evidence for the capability to combine ammonium ions and demonstrate a reduction in impedance values during the determination of ammonium when employing silver-based electrodes. This observation suggests that the modified electrodes possess enhanced conductivity, making them well-suited for various applications that necessitate efficient charge transfer.

The characterization of the Ag/poly-1-AAQ/CPE, as depicted in Fig. 3, revealed the presence of cylindrical needle-like structures covering the electrode surface with a resolution of 5 µm. Elemental mapping analysis, conducted using EDX analysis, demonstrated a distribution of carbon, nitrogen, and oxygen with high mass percentages of 60.19%, 31.55%, and 7.50%, respectively. This indicates the successful anodic polymerization of poly-1-aminoanthraquinone on the electrode surface, as evidenced by the formation of conductive polymer 1-AAQ in the form of cylindrical needles. The mass percentage of nitrogen and oxygen atoms on the electrode surface was found to be higher compared to that of Ag/CPE, confirming the incorporation of poly-1-aminoanthraquinone.

EDX data also revealed the presence of silver after chelation with active sites of poly-1-aminoanthraquinone, with a mass percentage of 0.76%. Although the mass percentage of silver was lower in Ag/poly-1-AAQ/CPE compared to Ag/CPE, this can be attributed to the wider dispersion of poly-1-AAQ on the electrode surface. This dispersion contributes to enhanced conductivity of the electrode, particularly during the impedimetric determination of ammonium, which is crucial for electrochemical impedance spectroscopy. Therefore, Ag/poly-1-AAQ/CPE was chosen as it combines the benefits of silver deposition and conductive polymer, leading to improved conductivity essential for EIS measurements. The successful deposition of silver on the electrode surface was confirmed through EDX data analysis. The presence of silver provides an appropriate candidate for chelation with ammonium in Tollen’s complex reagent, facilitating further analysis.

### Electrochemical impedance spectroscopy on modified electrodes

**Figure 4:**
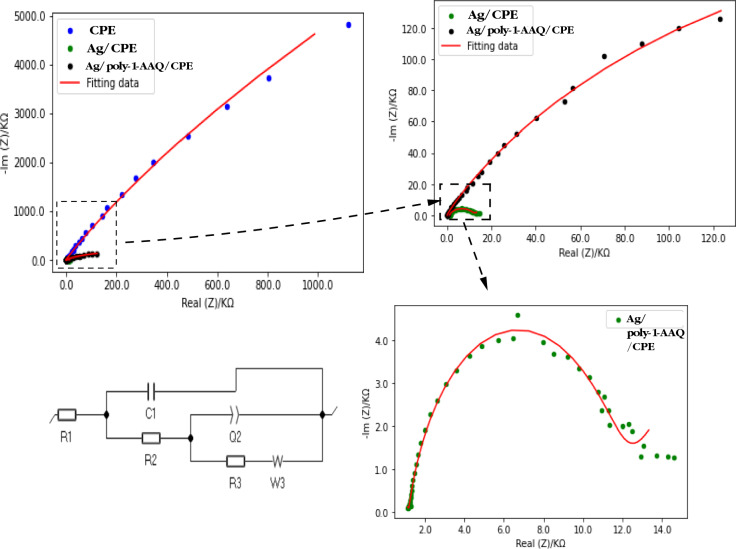
Experimental impedimetric spectra data obtained for a solution containing 100 µM NH_4_^+^ in 0.1 M Na_2_SO_4_, using CPE, Ag/CPE, and Ag/poly-1-AAQ/CPE. The fitting data was collected by applying the equivalent circuit shown in the bottom left. The magnifications of the images for Ag/CPE and Ag/poly-1-AAQ/CPE are displayed in the top right, while the magnification for the impedimetric data on Ag/CPE is shown in the bottom right.

In this study, the as-synthesized electrodes (bare CPE, Ag/CPE, and Ag/poly-1-AAQ/CPE) were characterized using EIS in a solution of 0.1 M Na_2_SO_4_ containing a concentration of 100 µM NH_4_^+^. The experimental EIS data showed best matching with the fitted data output from the simulated equivalent circuit. The equivalent circuit as shown in Fig. 4, composed of different components describe the properties of the electrochemical system. The equivalent circuit composed of six components: the solution resistance R1, the double layer capacitance C1, the capacitance of the film Q1, the film resistance and the double layer capacitance R2 and finally the charge transfer resistance R3 which is most important character. The charge transfer resistance is the most essential component because it belongs to the kinetic of the charge transfer reaction at the electrolyte–electrode surface, in case of analytical system, it indicates the analytical performance of ammonium on modified electrode. The Nyquist plots (Fig. 4) showed high impedimetric data ranging from thousands of KΩ for bare CPE to a few hundred KΩ for Ag/poly-1-AAQ/CPE, and even lower values in the range of a few KΩ for Ag/CPE. Notably, the Ag/CPE exhibited a large diameter semi-circle in the impedance data, indicating a low charge transfer resistance compared to the conductive polymer modified electrode Ag/poly-1-AAQ/CPE.

The charge transfer resistance values were found to be 87.37 × 10^3^ KΩ for bare CPE, 21 KΩ for Ag/CPE, and 535 Ω for Ag/poly-1-AAQ/CPE. These results demonstrate a significant reduction in charge transfer resistance for the modified electrodes, highlighting the superior performance of Ag/poly-1-AAQ/CPE for the impedimetric determination of ammonium ions.

The decrease in charge transfer resistance observed in the impedimetric measurements of ammonium on a silver-based modified electrode, specifically Ag/CPE, can be attributed to the reaction between silver on the modified electrode and ammonium, forming Tollen's complex. This reaction follows Eq. (1).

1 $$4{{NH}_{4}^{+}}+AgN{O}_{3}+{Ag}^{0} \to 2\left[Ag{\left({NH}_{3}\right)}_{2}\right]+{HNO}_{3}+3 {H}^{+}$$

The reaction involves the formation of zero-valent silver ions when a fixed potential of + 0.75 V is applied. These silver ions then react with ammonium ions present in the solution, as well as residual silver nitrate, resulting in the formation of diamine silver (I) complex. This complex formation directly affects the resistance of the modified electrode in the ammonium solution. In the case of Ag/poly-1-AAQ/CPE, the reduction of charge transfer resisatance indicates the feasibility of silver deposition within the poly-1-AAQ structure. This deposition allows for more accessible zero-valent silver, leading to the formation of a greater amount of diamine silver (I) complex. Consequently, the charge transfer resistance decreases. This reduction in charge transfer resistance is particularly significant when considering higher ammonium concentrations, as will be further discussed in subsection c.

### Analytical performance

EIS offers numerous advantages for establishing an analytical system as it considers different components of the electrochemical system. To evaluate the performance of the analytical system, the modified electrode Ag/poly-1-AAQ/CPE was tested with varying concentrations of ammonium in a working medium of 0.1 M Na_2_SO_4_. The EIS data was represented by Nyquist plots for concentrations ranging from 5 µM NH_4_^+^ to 200 µM NH_4_^+^, and the experimental data was fitted using an equivalent circuit.

The equivalent circuit comprised various components including charge transfer resistance (R3), solution resistance (R1), film resistance (R2), double layer capacitance (C1), and film capacitance (Q1). The film resistance is particularly influenced by the ionic strength of the electrolyte solution and the distance between the working electrode and auxiliary electrode. For the 0.1 M Na_2_SO_4_ solution, a relatively consistent value of R1, around 100 ± 10 Ω, was observed according to the simulated equivalent circuit. The charge transfer resistance is associated with the kinetics of charge transfer at the interface between the electrolyte and electrode. As the concentration of ammonium ions increased, the charge transfer resistance decreased, indicating more efficient electron transfer at the electrode–electrolyte interface. The concentration of ammonium significantly influences the charge transfer resistance by modulating the reaction kinetics and conductivity.

**Figure 5:**
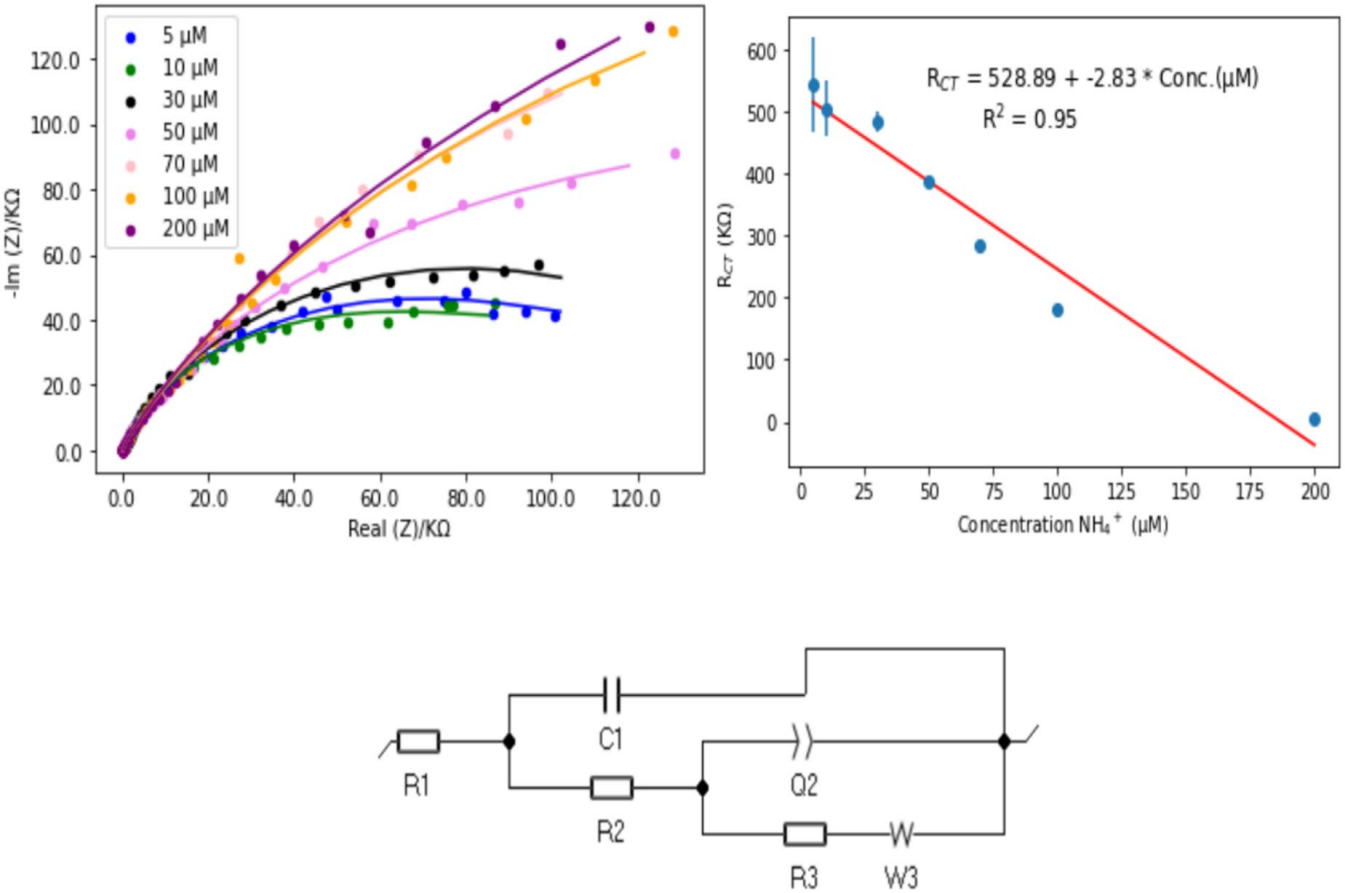
Impedimetric spectra data for various concentrations of NH_4_^+^ (ranging from 5 to 200 µM) in a solution of 0.1 M Na_2_SO_4_, using the Ag/poly-1-AAQ/CPE. The top left figure displays the experimental impedimetric data, along with the fitting data obtained using the equivalent circuit shown at the bottom. The top right section shows the calibration plot for the charge transfer values derived from the R3 component in the equivalent circuit, with a depicted linear equation ${R}_{CT}=528.89+\left(-2.83\right) \times Conc ({NH}_{4}^{+})$ and ${R}^{2}=0.95$.

This relationship was demonstrated by the linear correlation between the charge transfer values and ammonium concentrations in the regression plot (Fig. 5), with a good linearity represented by an R square value of 0.95. The linear calibration plot exhibited a sensitivity of 2.83 kΩ µM^−1^. The detection limit for the Ag/poly-1-AAQ/CPE in the impedimetric determination of ammonium was determined to be 3.3 µM NH_4_^+^. The limit of detection (LOD) was calculated as three times the standard deviation of blank responses (based on the standard deviation of 10 blank responses) divided by the slope of the calibration plot.

The modified electrode exhibited excellent analytical performance for the determination of ammonium concentrations, surpassing other electrochemical methods reported in the literature. Our method employs impedimetric analysis for the direct determination of ammonium in a slightly strong supporting medium (0.1 M Na_2_SO_4_). Impedimetric sensing offers distinct advantages over other electrochemical techniques, particularly due to its high sensitivity and ability to account for the complexity of the electrochemical sensor.

As EIS considers all components within the electrochemical analytical system, including the charge transfer resistance, it enhances the sensitivity of the analytical system. Table 1 presents the data for the linear range and limit of detection (LOD) values of various modified electrodes reported in the literature. Among the published electrode modification approaches, cyclic voltammetry exhibited a higher LOD value of 18.4 µM with an enzymatic sensor, while chronoamperometry showed a lower LOD value. Potentiometric techniques suffered from low sensitivity and lack of repeatability.

**Table 1:** Electrochemical determination of NH_4_^+^on different modified electrodes.

Electrode Construction	Technique	Linear range (µM)	LOD (µM)	References
GLDH/α-KG/NADH/MB/SPCE	CV (depending on NADH concentration)	18.4–184.0	18.4	^24^
GLDH-CS/diaphorase-CS/SPCE	Chronoamperometry	2.5–500	2.5	^25^
GLDH/Fe_3_O_4_/GR/CS/GC	DPV	0.4–2.0	0.08	^26^
CuO/ZnO NCs	Voltammetric (I–V method)	77.0–7.7 × 10^6^	8.9	^27^
Ag/CNT	DPV	200–1000	1	^28^
Ag/PAAQ/GC	SWV	0.55–500	0.17	^22^
Ag/PAAQ/MWCNTs/CPE	SWV	5–100	0.03	^10^
Ag/PAAQ/CPE	EIS	5–200	3.3	This work

In contrast, voltametric pulsed techniques such as differential pulse voltammetry and square wave voltammetry, which sample the current twice per pulse, increase sensitivity and reduce the capacitive current. Previous studies utilizing pulsed techniques demonstrated lower detection limits ranging from 0.03 µM to 1 µM NH_4_^+^. Although our EIS-based method achieved an LOD of 3.3 µM, it outperforms others in terms of its suitability for integration into autonomous prototypes. Additionally, the precision of the analytical system is highly favourable for impedimetric analysis in EIS, as it does not suffer from carry-over issues that can contaminate results in other electrochemical techniques.

Different instruments were employed to determine the concentration of ammonium were summarized in Table 2, including spectrophotometric, fluorometric, liquid chromatography, and electrochemistry. In the sequential flow analysis, the OPA reagent method was utilized, while liquid chromatography was developed for ammonium determination. LOD for these methods ranged from 0.001 to 0.3 µM NH_4_^+^. Another method employed in sequential flow analysis was the indophenol blue method, which utilized the phenol reagent and a long waveguide capillary flow cell to achieve an LOD of 0.005 µM NH_4_^+^. Additionally, the pH indicator bromothymol blue was applied in flow spectrophotometry, resulting in an LOD of 0.015 µM. For ammonium determination, electrochemical analytical techniques were also employed. Although these techniques offered slightly rational LODs, they provided advantages such as simplicity, ease of operation, and integration into autonomous deployable sensors for on-field monitoring. Notably, electrochemical impedance spectroscopy offered a straightforward and manageable sensing option. In addition, despite having a relatively higher LOD value compared to electrochemical methods that use pulsed techniques like SWV and DPV, the use of Electrochemical Impedance Spectroscopy (EIS) for ammonium determination is highly desirable. EIS is a non-destructive technique^29^ that provides valuable information about the electrochemical system, enabling researchers to understand its behaviour better.

**Table 2:** Comparison between different analytical techniques for the determination of NH_4_^+^ in water.

Technique	Chemistry	Linear range (µM)	LOD (µM)	References
Modified SIA (termed ABA)	OPA-Sulfide with Formaldehyde	0.005–25	0.001	^30^
SFA-LWCC	IPB-Phenol	0.005–10	0.0055	^31^
Programmable flow Spectrophotometric	pH-indicator bromothymol blue	0.028–55.6	0.015	^32^
LC with fluorescence detection	OPA-Sulfide	0.625–10	0.33	^33^
SWV-electrochemistry	Ag-PAAQ-MWCNT-CPE	5–100	0.03	^10^
EIS-electrochemistry	Ag-PAAQ-CPE	5–200	3.3	This work

*SIA* sequential injection analysis, *SFA-LWCC* segmented flow analyzer-liquid waveguide capillary cell, *LC* liquid chromatography.

**Figure 6:**
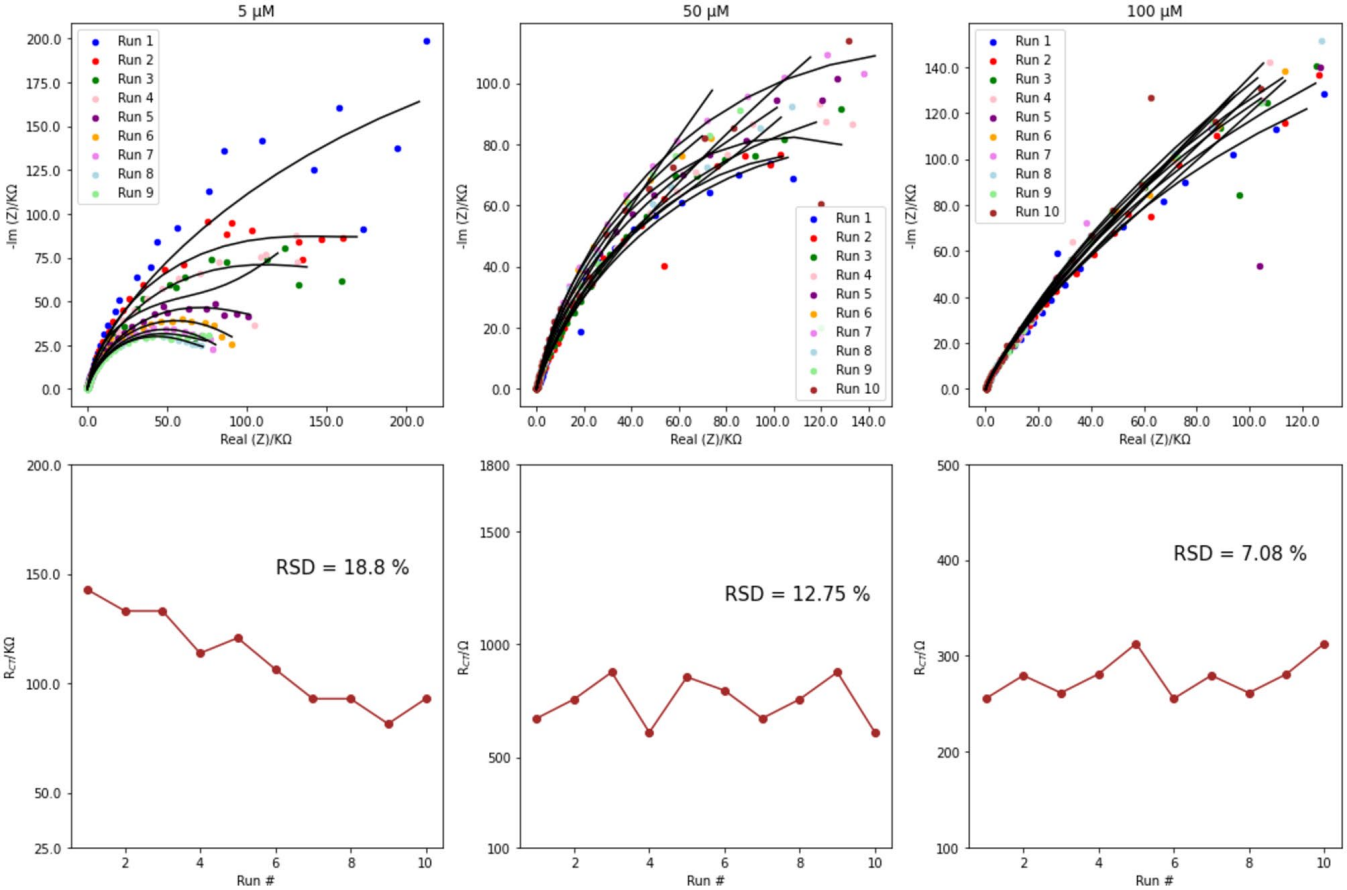
Nyquist plots illustrating the impedimetric data obtained for Ag/poly-1-AAQ/CPE in a solution of 0.1 M Na_2_SO_4_, with NH_4_^+^ concentrations of 5 µM, 50 µM, and 200 µM. The top figures display 10 repeated EIS measurements for each concentration. The bottom figures present the corresponding charge transfer resistance values for each NH_4_^+^ concentration, along with the RSD values. The RSD values were found to be 18.8% for 5 µM, 12.75% for 50 µM, and 7.08% for 200 µM.

To evaluate the analytical performance of the modified electrode for impedimetric determination of ammonium in water samples, we conducted multiple measurements to assess the repeatability of our sensor. The Ag/poly-1-AAQ/CPE was tested for 10 measurements at varying concentrations of ammonium: low, medium, and high ranges as shown in Fig. 6. In the impedimetric determination of 5 µM NH_4_^+^ in 0.1 M Na_2_SO_4_, the modified electrode exhibited a RSD of 18.8%, with an average value of 113 × 10^3^ Ω. As the concentration increased to 50 µM NH_4_^+^, the RSD decreased to 12.75%, with an average value of 764 Ω. Further reduction in RSD with a value of 7.08% was observed at a concentration of 200 µM NH_4_^+^, with an average value of 274 Ω. Interestingly, the RSD values were higher than those reported by Gibbons et al., which may be attributed to the extremely high charge transfer resistance values in the range of thousands of kΩ at low concentrations reflected in high standard deviation values and consequently RSD values, transitioning to the hundreds of Ω for moderate concentrations of NH_4_^+^.

### Evaluation of the modified electrodes

A method was employed to directly analyze ammonium in spiked samples, and its suitability was assessed using various measures. Three samples were prepared and analyzed using Ag/poly-1-AAQ/CPE for three measurements. The results obtained from these analyses are presented in Table 3. The recovery values ranged from 87.5 to 109% for concentrations of 5 µM, 10 µM, and 70 µM. While the recovery values for the assigned and obtained NH_4_^+^ concentration values were not ideal, there was minimal bias (systematic error) observed. To evaluate the bias between the assigned values and the measured values of NH_4_^+^, a paired t-test was conducted. For each sample, three analyses were performed, and a paired t-test was carried out with a degree of freedom of 2 and a significance level of 5% to determine the presence of systematic error (bias). For the concentration of 5 µM, no bias was detected as the calculated t-value (2.14) was less than the critical t-value with two tails (4.3). This result was also observed for the concentration of 10 µM, where the calculated t-value (1.53) was less than the critical t-value with two tails (4.3). However, for the concentration of 70 µM, a bias was identified to a small extent, as the calculated t-value (4.35) was greater than the critical t-value with two tails (4.3). The results demonstrate that our method has the capability to accurately detect NH_4_^+^ concentration in water samples, showing minimal systematic error for both low and high concentrations.

**Table 3:** Analysis of samples by Ag/poly-1-AAQ/CPE.

Assigned concentration (NH_4_^+^) (µM)	Measured concentration by Ag/poly-1-AQQ/CPE (µM)	Recovery (%)
5	4.4	88
10	8.75	87.5
70	76.32	109

## Conclusions

The modified electrode Ag/poly-1-AAQ/CPE was prepared using cyclic voltammetry and applied potential. The successful preparation of the electrode was confirmed through various characterization techniques such as SEM, EDX, and elemental mapping analysis. These analyses demonstrated the deposition of Ag on the electrode surface. The Ag/poly-1-AAQ/CPE was utilized for the impedimetric determination of ammonium in water samples with a concentration of 0.1 M Na_2_SO_4_. Our modified electrode exhibited a linear relationship between the values of charge transfer resistance and the concentrations of ammonium. Furthermore, it demonstrated good sensitivity, with a LOD value of 3.3 µM NH_4_^+^.In addition to its sensitivity, the modified electrode also displayed high precision, as indicated by the RSD values of 18.8%, 12.75%, and 7.08% for concentrations of 5 µM, 50 µM, and 200 µM NH_4_^+^, respectively. Moreover, no significant bias was observed between the assigned values and the measured values for three samples with concentrations of 5 µM, 10 µM, and 70 µM NH_4_^+^. Additionally, the recovery values obtained were within an acceptable range.

## Data Availability

The datasets used and/or analysed during the current study available from the corresponding author on reasonable request.
